# Magnetic Bead-Based Serum Peptidome Profiling in Patients with Gestational Diabetes Mellitus

**DOI:** 10.1155/2015/586309

**Published:** 2015-05-18

**Authors:** Tingting Ai, Feng Chen, Shaonan Zhou, Jieni Zhang, Hui Zheng, Yanheng Zhou, Wei Hu, Xiaofei Liu, Li Li, Jiuxiang Lin

**Affiliations:** ^1^Department of Orthodontics, School of Stomatology, Peking University, No. 22 Zhongguancun South Road, Haidian District, Beijing 100081, China; ^2^Central Laboratory, School of Stomatology, Peking University, Beijing 100081, China; ^3^Beijing Bioyong Technologies Inc., Beijing 100085, China; ^4^Clinical Laboratory, Beijing Haidian Maternal & Child Health Hospital, Beijing 100080, China

## Abstract

Gestational diabetes mellitus (GDM) is a frequent medical condition during pregnancy. Early diagnosis and treatment of GDM are crucial for both the mother and the baby. In the present study, we aimed to identify specific biomarkers to assist in the early detection of GDM and give some clues to the possible causes of GDM by comparing serum peptide profile differences between GDM patients and healthy controls. Matrix-assisted laser desorption/ionization time-of-flight mass spectrometry (MALDI-TOF MS) was used in combination with weak cation exchange magnetic bead (WCX-MB). Levels of four peptides (4418.9, 2219.7, 2211.5, and 1533.4 Da) were significantly different. Interestingly, three of them (4418.9, 2211.5, and 1533.4 Da) were identified when GDM patients with two degrees of glucose intolerance were compared. Additionally, peptides 2211.5 and 1533.4 Da showed a decreasing trend as glucose intolerance increased, while peptide 4418.9 Da exhibited the reverse tendency. In conclusion, our study provides novel insights into the altered serum peptide profile of GDM patients. The specific candidate biomarkers may contribute to the development of GDM.

## 1. Introduction

Gestational diabetes mellitus (GDM) is a frequent medical condition during pregnancy and is defined as carbohydrate intolerance that begins or is first recognized during gestation. Women with GDM have an increased risk of complications in both themselves and the baby during pregnancy and birth and even later in life, such as future development of diabetes, obesity, and metabolic syndrome, which can seriously affect long-term quality of life [1]. Hence, an early diagnosis of GDM is crucial for preventing the occurrence and development of these diseases.

The main diagnostic test for GDM is oral glucose tolerance test (OGTT). However, this test is performed during the second and third trimesters of pregnancy, which may delay the optimal timing for treatment. In addition, OGTT is relatively slow and inconvenient as it needs to draw the blood from patients three times to establish the diagnosis. What is more, the diagnostic criteria of the test have changed several times over the years. Different organizations have adopted different cutoff points, suggesting difficulties in distinguishing GDM [2].

Serum constantly perfuses tissues and has a high protein content, with many of these proteins being released and secreted from cells and tissues. Therefore, the characterization of the thousands of serum proteins/peptides will enable the discovery of reliable useful biomarkers, which could serve to improve early disease detection. Recently, matrix-assisted laser desorption/ionization time-of-flight mass spectrometry (MALDL-TOF MS), one of the rapidly developing mass spectrometry- (MS-) based proteomic methods, has been widely applied to screen specific biomarkers from serum, saliva, and urine according to their mass-dependent velocities (*m/z*) due to its high sensitivity, relative convenience, and potential clinical applications [3].

As low-molecular-weight (LMW) region of the blood proteome is a treasure trove of diagnostic information and likely reflects the state of disease [4–6], purifying the LMW peptides from serum which has a high dynamic concentration range of constituent protein/peptide is an essential step. Compared with 2D-PAGE separation, which is problematic in detecting proteins that are smaller than 10 kDa, WCX magnetic bead used in our study could cover a range of 1–10 kDa [7]. The lower molecular weight range may contain peptides and proteolytic fragments that are abnormally released from cells in response to a disease.

MALDL-TOF MS combined with WCX-MB is a promising diagnostic and has been successfully applied to cancer diagnosis [8–11]; however, few studies using this technique investigated the discriminating protein profiles in the serum of women with GDM. Our study compared peptide expression profiles of serum samples from patients with and without GDM by MALDI-TOF MS-based peptidome analysis in combination with WCX magnetic beads, aiming to identify a panel of differentially expressed specific peptides to discuss possible causes and facilitate the early diagnosis of GDM.

## 2. Materials and Methods

### 2.1. Ethics Statement

This study was approved by Beijing Haidian Maternal & Child Health Hospital Biomedical Ethics Committee. All subjects signed an informed consent prior to the start of the study.

### 2.2. Subjects

Serum samples were collected from 24-week pregnant women that visited Beijing Haidian Maternal & Child Health Hospital in November 2012. All 21 study subjects were systemically healthy, and those at a high risk of GDM, such as a history of diabetes before conception, obesity, or miscarriage, were excluded.

Subjects were divided into two groups according to the diagnostic criteria of OGTT recommended by the International Association of the Diabetes in Pregnancy Study Group (IADPSG) [12]: healthy pregnant women and patients with GDM. The criteria used were as follows: healthy controls: fasting glucose < 5.1 mmol/L: 1 h result < 10 mmol/L and 2 h result < 8.5 mmol/L; GDM1: 1 h result of glucose ≥ 10 mmol/L and 2 h result ≤ 8.5 mmol/L; GDM2: 1 h result of glucose ≥ 10 mmol/L and 2 h result ≥ 8.5 mmol/L. Both GDM1 and GDM2 were patients with impaired glucose tolerance but different degrees of severity. According to the differences of the OGTT results, the GDMs and healthy controls were compared using 1 h serum and then GDM1 and GDM2 were further compared using 2 h samples.

The mean age and OGTT results of each group are shown in Table 1.

### 2.3. Serum Collection and Processing

All serum samples came from residual serum following an OGTT. In addition to 1 h postprandial serum of all subjects, 2 h serum samples of patients with GDM were also collected. The blood samples were allowed to separate into layers at 4°C for 24 h. The supernatants from each sample were obtained and stored at −80°C until further analysis.

### 2.4. Reagents and Instruments

The WCX magnetic bead kit (SPE-C; Bioyong Tech, Beijing, China), alpha-cyano-4-hydroxycinnamic acid (HCCA), MALDI-TOF MS (Bruker BioSciences, Bremen, Germany), 100% ethanol (chromatographic grade), and 100% acetone (chromatographic grade) were freshly prepared.

### 2.5. Serum Pretreatment with WCX Magnetic Bead

The low-molecular-weight peptides from the serum samples were purified and isolated using WCX magnetic bead according to the manufacturer's protocol (ClinProtTM, Bruker Daltonics) which was described in previous study [10]. After binding, washing, and eluting, the eluted peptide samples were transferred to a clean 0.5 mL sample tube for further analysis by MS.

### 2.6. MALDI-TOF MS Analysis

Five microliters of HCCA substrate solution (0.4 g/L, dissolved in acetone and ethanol) and 0.8–1.2 *μ*L of the eluted peptide samples were mixed. Then, 0.8–1.2 *μ*L of this mixture was applied to a metal target plate and dried at room temperature. Finally, the prepared sample was analyzed by MALDI-TOF MS. A range of 1,000–10,000 Da peptide molecular weights was collected, and 400 shots of laser energy were used. Peptide mass fingerprints were obtained by accumulating 50 single MS signal scans. The serum samples collected from each patient were analyzed serially for three times by MALDI-TOF MS. The mean values of each sample were used for statistical analysis.

### 2.7. Identification of Protein Biomarker by Nano-LC/ESI-MS/MS

The sequences of differentially expressed peptides between women with GDM and healthy controls were identified using a nanoliquid chromatography-electrospray ionization-tandem mass spectrometry (nano-LC/ESI-MS/MS) system consisting of an ACQUITY UPLC system (Waters) and a LTQ Orbitrap XL mass spectrometer (Thermo Fisher) equipped with a nano-ESI source. Firstly, the peptide solutions were loaded to a C18 trap column (nanoACQUITY) (180 *μ*m × 20 mm × 5 *μ*m (symmetry)) at a flow rate of 15 *μ*L/min. Then, the desalted peptides were enriched by C18 analytical column (nanoACQUITY) (75 *μ*m × 150 mm × 3.5 *μ*m (symmetry)) with the flow rate of 400 nl/min. Thirdly, the mobile phases A (5% acetonitrile, 0.1% formic acid) and B (95% acetonitrile, 0.1% formic acid) were used for analytical columns. The gradient elution profile was 5%B-50%B-80%B-80%B-5%B-5%B in 100 min. The MS instrument was operated in a data-dependent model. The range of full scan was 400–2000* m/z* with a mass resolution of 100,000 (*m/z* 400). The eight most intense monoisotope ions were the precursors for collision induced dissociation. MS/MS spectra were limited to two consecutive scans per precursor ion followed by 60 s of dynamic exclusion.

The obtained chromatograms were analyzed with BioworksBrowser 3.3.1 SP1 and the resulting mass lists were used for database search using Sequest (IPI Human (3.45)). Parameters for generating peak list were as follows: parent ions and fragment mass relative accuracy were set at 50 ppm and 1 Da, respectively.

### 2.8. Statistical Analysis

The *t*-test and *w*-test were used to compare peptide levels in serum samples from the two groups. Data were analyzed using the BioExplorer statistical package (Bioyong Tech) [13, 14]. A *P* value < 0.05 was considered to indicate statistical significance.

## 3. Results

**Figure 1:**
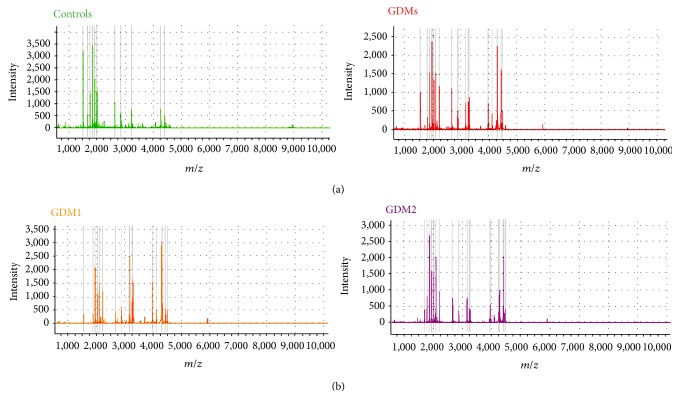
Complete mass spectra in the range of 1000–10000 Da, demonstrating the peptide fingerprints of the serum sample from a single patient in each group* m/z*, mass-to-charge ration. (a) GDMs (red curve) and controls (green curve) in the first comparison. (b) GDM1 (orange curve) and GDM2 (purple curve) in the second comparison.

The entire mass spectra of serum peptide samples from 21 subjects were obtained by MALDI-TOF MS with WCX-MB (Figure 1). Peaks in the serum peptidome fingerprints were featured in each patient by presenting the maximum intensity within a certain* m/z* range.

**Figure 2:**
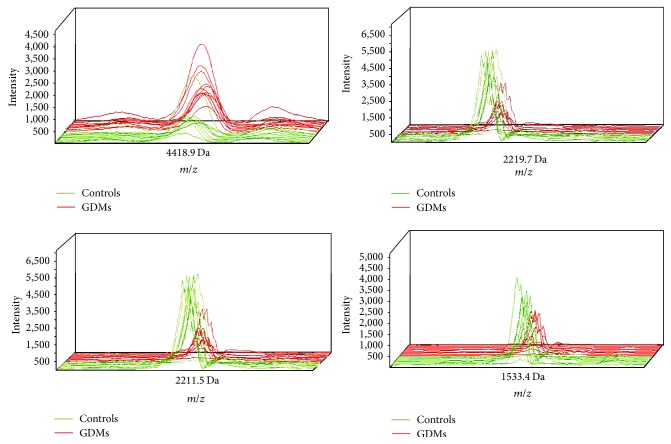
Three-dimensional* m/z* ratio-intensity maps showed the peak intensity of peptides at 4418.9, 2219.7, 2211.5, and 1533.4 Da, which had extremely significant difference between GDMs (red curve) and controls (green curve).

**Figure 3:**
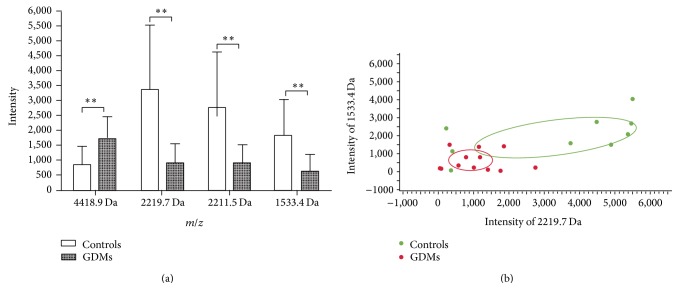
(a) Column view of the mass spectra from the two groups, showing that the peak intensity at 2219.7, 2211.5, and 1533.4 Da of GDMs is significantly lower, whereas the intensity at 4418.9 Da of GDMs increased evidently (^**^ *P* < 0.01). (b) Scatter plots of the GDMs (red dot) and controls (green dot) established by the combination of peptides 2219.7 and 1533.4 Da, showing a well-discriminated fitting shape of the curve.

A total of 40 peptide mass peaks ranging from 1,000 to 10,000 Da were detected from* m/z* spectra when GDM patients and healthy controls were compared. Peak intensities of four peptides (4418.9, 2219.7, 2211.5, and 1533.4 Da; Table 2(a)) differed significantly (Figure 2). The mass peaks of peptides 1533.4, 2211.5, and 2219.7 Da were lower in the GDMs, whereas the peak of peptide 4418.9 was higher (Figure 3(a)). In addition, peptides 1533.4 and 2219.7 established the most-fitted curves of any two peptide combinations (Figure 3(b)). The curves were well-separated for the samples of these two groups, indicating a satisfactory fitting result.

**Figure 4:**
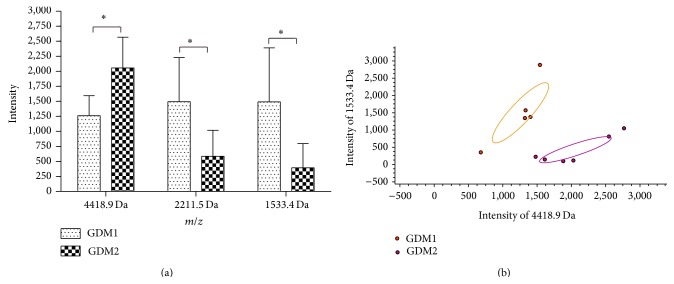
(a) Column view of the mass spectra from the two groups, showing that the peak intensity at 2211.5 and 1533.4 Da of GDM2 was significantly lower, whereas the intensity at 4418.9 Da of GDM2 was higher (^*^ *P* < 0.05). (b) Scatter plots of the GDM1 (orange dot) and GDM2 (purple dot) established by the combination of peptides 4418.9 and 1533.4 Da, showing a well-discriminated fitting shape of the curve.

When the 2 h serum samples of GDM1 (less severe glucose intolerance) and GDM2 (more severe glucose intolerance) patients were compared, the intensities of peaks 1533.4, 2211.5, and 4418.9 Da showed a significant difference, which were also detected during the comparison of healthy controls and GDMs (Table 2(b)). The intensities of peptides 1533.4 and 2211.5 of GDM2 patients were lower than GDM1, whereas the intensity of peptide 4418.9 Da was increased in GDM2 patients (Figure 4(a)). Furthermore, peptides 1533.4 and 4418.9 established a well-distinguished fitting curve that facilitated separation of GDM1 and GDM2 (Figure 4(b)).

What is more, the peptides at 1533.4, 2211.5, and 2219.7 were predicted to be clusterin (CLU) precursor, isoform 1 of fibrinogen alpha chain (FGA) precursor, and apolipoprotein C II (APOC II) precursor using LTQ Orbitrap MS detection. The sequencing results of the three identified peptides were shown in Table 2(c).

## 4. Discussion

With the alterations in the hormonal milieu during pregnancy, carbohydrate and lipid metabolism change. However, in women who develop GDM, these metabolic changes are progressive and may be in an extreme state [15]. As a large number of peptides and proteins in the serum were involved in the dysfunctional metabolism, we aimed to identify some meaningful proteomic biomarkers in the serum of patients with GDM through serum peptide profile analysis.

In the present study, we successfully found the differential expressed peptides (4418.9, 2219.7, 2211.5, and 1533.4 Da) between normal pregnant women and women with GDM by MALDI-TOF MS combined with WCX magnetic beads. Interestingly, peak intensities of 1533.4, 2211.5, and 4418.9 Da also showed a significant difference when the two different degree GDM groups were compared. Furthermore, the trends of the three peptides changed as the degree of glucose intolerance increased, indicating that these peptides are closely related to GDM and can be used to evaluate its severity (Figures 3(a) and 4(a)).

The sequence identifications of the detected peptides have led to interesting speculations. The 1533.4 Da peptide was predicted to be clusterin precursor. The major function of secreted clusterin (sCLU) is protecting cells from the deleterious effects of reactive oxygen species (ROS) [16, 17] and is thus involved in various physiologic processes and many pathologic conditions that are relevant to ROS, including aging, cancer, and neurological disorders [16, 18–20]. ROS can also lead to both insulin resistance and pancreatic *β*-cell dysfunction and thus appears to play an important part in the pathophysiology of diabetes [21–23]. Additionally, late pregnancy can result in an increase in oxidative stress [24], which is higher in women with GDM [25, 26]. Our results, in combination with these studies, allow us to deduce that decreased clusterin, which is insufficient to protect cells from oxidative stress owing to pregnancy, may lead to GDM. Another function of clusterin is an apolipoprotein function on the high-density lipoprotein (HDL) particle (also called apolipoprotein J) [27]. In addition, peptide 2219.7, which was markedly decreased in GDMs, was predicted to be apolipoprotein C II (APOC II) precursor. Both of the proteins play a key role in the lipid metabolism and maybe associated with the development of the GDM. However, a peptide sequence usually does not identify a specific protein. Firstly, the sequence of the peptide acquired by experiment is usually a part of the entire sequence of the protein; the left parts depend on the database to fill in. What is more, any co- and posttranslational modification of amino acids could lead to a distinctive functional protein. In addition, the origins of the low-molecular-weight peptides extracted by the magnetic beads are complicated. They could be proteolytic fragments of circulating proteins or secreted peptides in serum.

Formal classic testing for gestational diabetes is oral glucose tolerance test (OGTT) and is usually done between 24 and 28 weeks of gestation. However, this method is not suitable for the early part of pregnancy and thus may miss the optimal interventions to control the development of GDM. Recently, a number of biomarkers have been identified for early detection of GDM, such as sex hormone binding globulin (SHBG) [28] and placental growth hormone (PGH) [29], which are related to pregnancy, as well as free fatty acids [30], adiponectin [31, 32], and follistatin-like-3 [33] which correlate with obesity and lipid metabolism. Most of these studies identified or verified one certain biomarker in the serum samples using the classical detection method such as ELISA. The molecular weight of these biomarkers tends to be larger than 10 kDa. In the present study, we used MALDI-TOF MS analysis combined with beads fraction, one of the proteomic methods, to separate low-molecular-weight (<10 kDa) peptides that were differentially expressed in the serum of GDM patients, aiming to detect more meaningful biomarkers comprehensively and to provide a novel tool distinguishing the patients with GDM. One of the biomarkers we identified was peptide 1533.4 that was predicted to be clusterin precursor. This protein not only protects cells from oxidative stress that related to pregnancy, but also plays an essential part in the lipid metabolism, hinting its close relationship with the development of GDM. Although these biomarkers were detected from 24-week pregnant women when the diagnosis of GDM has been made, they tended to be stable and may play a key role in the development of GDM constantly. In order to apply these identified peptides to the early detection of GDM, we will collect a dataset of pregnant women early in the first trimester for further validation of the biomarkers we identified in subsequent work.

In another study that also investigated differential expression of proteins in the circulation of patients with GDM using proteomic methods [34], the molecular weights of differential peaks (9122 Da, 9412 Da, 9701 Da, 17 105 Da, 17 222 Da, and 17 350 Da) were out of the range we detected, which may be attributed to the different protein extracting methods. Both studies were performed by mass spectrometry technology; however, anion exchange (Q10) ProteinChip Array was selected in Kim's study, whereas our study used weak cation exchange (WCX) magnetic bead as a proteome fraction tool. The Q10 chips that are usually combined with SELDI TOF MS contain quarternary ammonium groups, providing a cationic surface that can participate in electrostatic interactions with negatively charged aspartic acid and glutamic acid residues. WCX-MB that is often used in MALDI-TOF MS is based on superparamagnetic microparticles with negatively charged functional groups at their surface enabling cation exchange chromatography. Therefore, the opposite charged surfaces led to the different ranges of peptides detected. In MALDI-TOF technology, there are several kinds of magnetic bead available; however, some studies revealed that WCX-MB could get more peptides than others, especially in the low-molecular-weight range [35, 36].

In conclusion, using magnetic bead-based MALDI-TOF MS, we identified proteomic biomarkers that were not only significantly different in patients with GDM, but also closely related with increasing glucose intolerance. Our results provide a novel insight into the altered serum peptide profile during the development of GDM. The candidate biomarkers may contribute to the pathological process of GDM.

## Figures and Tables

**Table 1:** Characters and information of subjects.

Group	Sample size	Age		OGTT results (mmol/L)	
		Mean	SD	Mean	SD
Control	10	31.0	3.1623	FPG^a^: 4.46 1 h: 7.17	0.4266 1.2724
				2 h: 6.37	0.7312
GDM1	5	32.8	2.2804	FPG: 4.79 1 h: 10.43	0.5007 0.2327
				2 h: 6.75	0.7669
GDM2	6	32.7	2.1603	FPG: 5.51 1 h: 11.10	0.5954 0.9197
				2 h: 9.99	1.1404

^a^FPG: fasting plasma glucose.

**(a):** 

Mean *m*/*z* value	*P* value	Tendency^b^
4418.9	0.005	↑
2219.7	0.003	↓
2211.5	0.006	↓
1533.4	0.007	↓

^b^Tendency ↑: the intensity of *m*/*z* values of GDMs is higher than the controls; ↓: the intensity of GDMs is lower than the controls.

**(b):** 

Mean *m*/*z* value	*P* value	Tendency^c^
4418.9	0.014	↑
2211.5	0.029	↓
1533.4	0.022	↓

^c^Tendency ↑: the intensity of *m*/*z* values of GDM2 was higher than the GDM1; ↓: the intensity was lower in GDM2.

**(c):** 

Mean *m*/*z* value	Peptide sequence	Identified peptide
4418.9	—	Unknown peptide
2219.7	A.MSTYTGIFTDQVLSVLKGEE. -	Apolipoprotein C II precursor
2211.5	Y.KMADEAGSEADHEGTHSTKRG.H	Isoform 1 of fibrinogen alpha chain precursor
1533.4	R.RPHFFFPKSRIV.R	Clusterin precursor
